# c-Myc Is Required for Maintenance of Glioma Cancer Stem Cells

**DOI:** 10.1371/journal.pone.0003769

**Published:** 2008-11-20

**Authors:** Jialiang Wang, Hui Wang, Zhizhong Li, Qiulian Wu, Justin D. Lathia, Roger E. McLendon, Anita B. Hjelmeland, Jeremy N. Rich

**Affiliations:** 1 Department of Surgery, Duke University, Durham, North Carolina, United States of America; 2 Department of Pharmacology and Cancer Biology, Duke University, Durham, North Carolina, United States of America; 3 Department of Pathology, Duke University, Durham, North Carolina, United States of America; 4 Department of Medicine, Duke University, Durham, North Carolina, United States of America; 5 Preston Robert Tisch Brain Tumor Center, Duke University Medical Center, Durham, North Carolina, United States of America; 6 Department of Stem Cell Biology and Regenerative Biology, Cleveland Clinic, Cleveland, Ohio, United States of America; University of Helsinki, Finland

## Abstract

**Background:**

Malignant gliomas rank among the most lethal cancers. Gliomas display a striking cellular heterogeneity with a hierarchy of differentiation states. Recent studies support the existence of cancer stem cells in gliomas that are functionally defined by their capacity for extensive self-renewal and formation of secondary tumors that phenocopy the original tumors. As the c-Myc oncoprotein has recognized roles in normal stem cell biology, we hypothesized that c-Myc may contribute to cancer stem cell biology as these cells share characteristics with normal stem cells.

**Methodology/Principal Findings:**

Based on previous methods that we and others have employed, tumor cell populations were enriched or depleted for cancer stem cells using the stem cell marker CD133 (Prominin-1). We characterized c-Myc expression in matched tumor cell populations using real time PCR, immunoblotting, immunofluorescence and flow cytometry. Here we report that c-Myc is highly expressed in glioma cancer stem cells relative to non-stem glioma cells. To interrogate the significance of c-Myc expression in glioma cancer stem cells, we targeted its expression using lentivirally transduced short hairpin RNA (shRNA). Knockdown of c-Myc in glioma cancer stem cells reduced proliferation with concomitant cell cycle arrest in the G_0_/G_1_ phase and increased apoptosis. Non-stem glioma cells displayed limited dependence on c-Myc expression for survival and proliferation. Further, glioma cancer stem cells with decreased c-Myc levels failed to form neurospheres *in vitro* or tumors when xenotransplanted into the brains of immunocompromised mice.

**Conclusions/Significance:**

These findings support a central role of c-Myc in regulating proliferation and survival of glioma cancer stem cells. Targeting core stem cell pathways may offer improved therapeutic approaches for advanced cancers.

## Introduction

The emerging cancer stem cell model suggests that tumors are organized in a hierarchy with a subpopulation of cancer stem cells responsible for tumor maintenance and progression. Cancer stem cells are highly tumorigenic and phenocopy the original tumors in rodent xenograft models. Depletion of the cancer stem cell population greatly impairs the potential to initiate xenograft tumor formation of the bulk tumors [1], [2]. The cancer stem cell population also contributes to solid tumor angiogenesis [3], metastasis [4], and resistance to chemotherapy and radiotherapy [3], [5], [6], [7], [8], [9]. While this model has been validated in a growing list of haematopoietic and solid tumors, the molecular signaling pathways orchestrating the biology of cancer stem cells remain to be elucidated.

The c-Myc oncoprotein has been extensively studied for its instrumental role in proliferation and growth of normal and neoplastic cells. Deregulated c-Myc is found in diverse human tumors and is often associated with advanced malignancy and poor prognosis [10]. As c-Myc has been recently recognized as an important regulator of stem cell biology, it may serve as a link connecting malignancy and “stemness” [11]. In either normal or transformed cells, c-Myc alone activates an embryonic stem cell-like transcriptional module, which strongly correlates with tumor metastasis and mortality [12]. Ectopic c-Myc expression in transformed human keratinocytes dramatically increases the cancer stem cell fraction and enhances tumorigenicity [12]. Introduction of c-Myc with other transcription factors (including Oct3/4, Sox2, and Klf4) generates induced pluripotent stem (iPS) cells from differentiated cells [13]. Excluding c-Myc from this combination without eliminating endogenous c-Myc expression, drastically reduces the efficiency of iPS cell production [13], [14], [15], [16]. While all of these data suggest a role for c-Myc in maintaining stem cells, other functions of c-Myc in regulating stem cell biology have also been described. Conditional knockout of c-Myc in mouse bone marrow does not prevent proliferation or self-renewal of haematopoietic stem cells [17]. It rather results in accumulation of haematopoietic stem cells in bone marrow, suggesting that c-Myc specifically controls the interaction between haematopoietic stem cells and their niches. Additionally, over-expression of c-Myc-estrogen receptor fusion protein in human epidermal stem cells drives differentiation rather than proliferation [18], [19].

Because of the recognized functions of c-Myc in both normal stem cell biology and neural malignancy, we investigated the role of c-Myc in human glioma cancer stem cells. Gliomas are the most common primary intrinsic tumor type of the central nervous system. High grade gliomas (World Health Organization grades III and IV) are among the most lethal human malignancies [20]. In glioma, c-Myc expression correlates with the grade of malignancy [21]. Expression of c-Myc driven by the glial fibrillary acidic protein (GFAP)-promoter in developing mouse astroglia induces tumors that resemble human glioblastoma multiforme [22]. In this mouse model, the tumor mass contains fast dividing subpopulation that express c-Myc and relatively quiescent tumor cells that lack c-Myc expression [22]. We have now determined that the glioma cancer stem cells expressed higher levels of c-Myc relative to matched non-stem tumor cells and the activity of c-Myc is required for proliferation, growth, and survival of glioma cancer stem cells. Loss of c-Myc abolished xenograft formation by glioma cancer stem cells, underscoring a key role of c-Myc in glioma cancer stem cell maintenance.

## Results

### Glioma cancer stem cells express high levels of c-Myc

**Figure 1:**
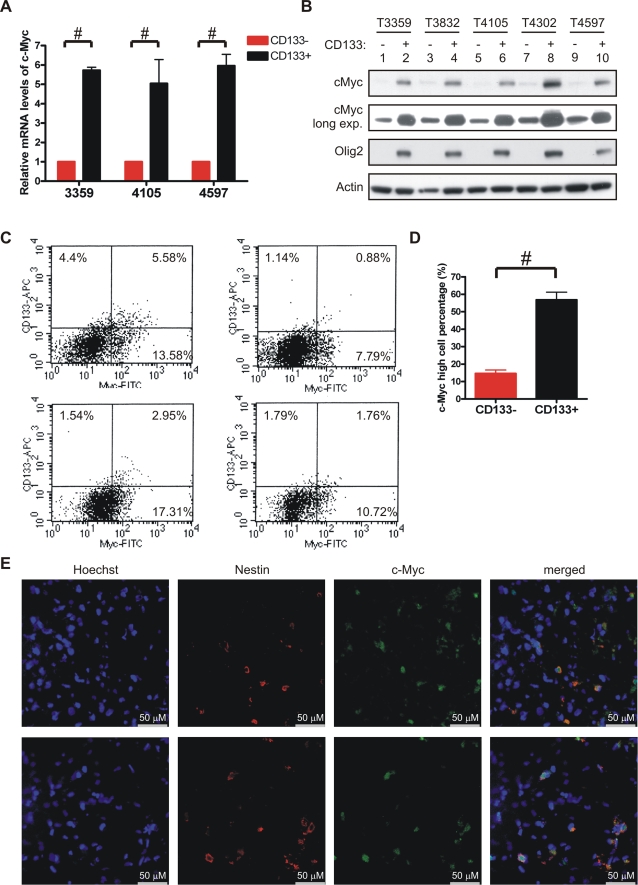
c-Myc is highly expressed in glioma cancer stem cells. (A) CD133− and CD133+ cells were isolated from glioma surgical biopsy specimens passaged short-term in immunocompromised mice and briefly cultured. Total RNA was isolated from both CD133− and CD133+ cells. cDNA was prepared by reverse transcription. Expression of c-Myc was then determined by quantitative real-time PCR and normalized to β-actin and HPRT1. Relative mRNA levels of c-Myc in CD133− cells were assigned a value of 1. Data are represented as mean±S.E.M in this and all subsequent graphs (#: p<0.001). (B) Total cellular lysates were resolved by SDS-PAGE. Protein levels of c-Myc and Olig2 were determined by immunoblotting. Actin was blotted as the loading control. (C) Glioma cells were isolated directly from human surgical biopsy specimens, fixed in 4% paraformaldehyde following dissociation, labeled with anti-CD133-APC and anti-c-Myc-FITC, and subjected to FACS analysis. (D) Percentage of cells expressing high levels of c-Myc within either the CD133− fraction or the CD133+ fraction was demonstrated (#: p<0.001). (E) Sections of freshly frozen human glioma surgical biopsy specimens were fixed and co-stained for c-Myc (green) and Nestin (red). Nuclei were counterstained with Hoechst 33342. Representative images (630×) were demonstrated.

To investigate the biological functions of c-Myc in regulation of glioma cancer stem cells, we first determined expression of c-Myc in these cells. Short term cultures enriched or depleted for cancer stem cells were derived from human brain tumor specimens using CD133 selection and characterized as we have previously demonstrated [9]. Quantitative real-time PCR revealed that CD133+ glioma cells expressed higher c-Myc mRNA levels than matched CD133− glioma cells (Figure 1A). The differential mRNA levels translated into higher levels of c-Myc protein in CD133+ glioma cells than matched CD133− cells (Figure 1B). Consistent with prior reports [23], [24], the cancer stem cell-enriched fractions also expressed high levels of Olig2, a marker of adult neural multipotent progenitors (Figure 1B). To directly measure the expression of c-Myc in human brain tumors, we further evaluated c-Myc expression by flow cytometry in both CD133+ and CD133− fractions of glioma cells acutely isolated from human surgical biopsy specimens without *in vitro* culture (Figure 1C). Approximately half of the CD133+ cells were high in c-Myc expression, whereas nearly 90% CD133− cells expressed low levels of c-Myc (Figure 1D). We further examined c-Myc expression in glioma cancer stem cells using sections generated from acutely frozen human glioma surgical specimens. Because co-staining with CD133 was not compatible with antigen retrieval method required for c-Myc staining, we co-stained c-Myc with another neural stem cell marker Nestin. Greater than 90% of Nestin positive glioma cells were also c-Myc positive (Figure 1E). These data suggest that c-Myc is highly expressed in glioma cancer stem cells.

### c-Myc regulates cell cycle progression and proliferation of glioma cancer stem cells

**Figure 2:**
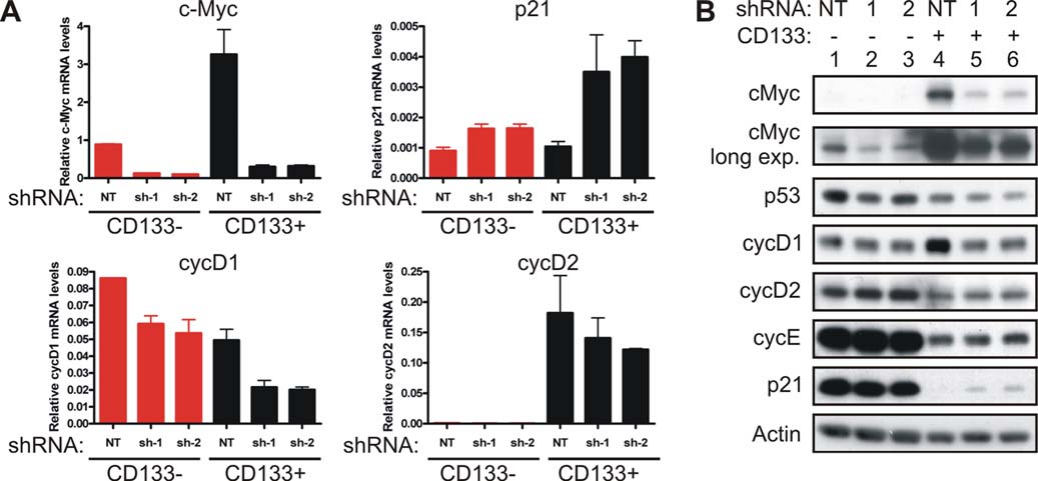
c-Myc modulates cell cycle regulators of glioma cancer stem cells. (A) Early passage (<P5) T3359 glioblastoma CD133− or CD133+ cells were infected by lentivirus directing expression of either non-targeting shRNA (NT) or c-Myc specific shRNAs (sh-1 and sh-2). The mRNA levels of c-Myc, p21^WAF1/CIP1^, cyclin D_1_ (cycD1) and cyclin D_2_ (cycD2) were determined by quantitative real-time PCR 3 days after infection. (B) Protein levels of c-Myc, p53, cyclin D_1_, cyclin D_2_, cyclin E and p21^WAF1/CIP1^ were determined by immunoblotting.

**Figure 3:**
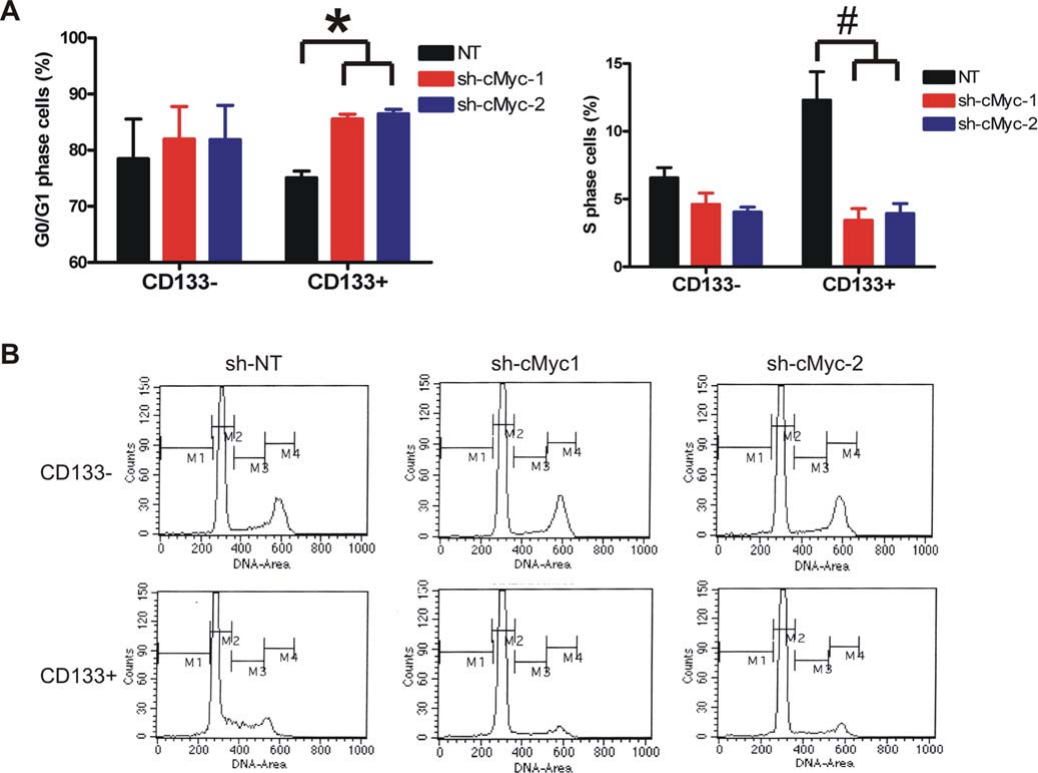
c-Myc induces cell cycle arrest of glioma cancer stem cells. (A, B) Early passage (<P5) T3359 glioblastoma CD133− or CD133+ cells were infected by lentivirus directing expression of either non-targeting shRNA (NT) or c-Myc specific shRNAs (sh-1 and sh-2). Three days after infection, cells were fixed in 75% ethanol and labeled with 10 µg/ml propidium iodide. Cell cycle distribution was determined by flow cytometry. (A) Data were generated from three independent experiments. *, p<0.05; #, p<0.001 with one-way ANOVA comparison of the control groups to the corresponding c-Myc knockdown groups. (B) Representitive FACS plots are displayed. M1-sub G_0_ phase; M2-G_0_/G_1_ phase; M3-S phase; M4-G2/M phase.

c-Myc plays a key role in regulating cellular proliferation by controlling the expression of cell cycle proteins. To interrogate the role of c-Myc in the cell cycle of glioma cancer stem cells, we targeted c-Myc expression by infection with lentivirus expressing shRNA specific to c-Myc. Two different shRNAs efficiently decreased c-Myc expression in both CD133− and CD133+ glioma cells as shown by quantitative real-time PCR and immunoblotting (Figure 2A and 2B). Similar results were found in another tumor specimen (T4597, data not shown). Depletion of c-Myc significantly reduced the S phase cell population with concomitant increase of the G_0_/G_1_ cell population in CD133+ cells (p<0.001, Figure 3). In contrast, CD133− cells displayed a lower rate of proliferation with a smaller S phase population than matched CD133+ cells (Figure 3 and data not shown), and the cell cycle progression in CD133− cells was not significantly altered by knockdown of c-Myc. Regulation of the cell cycle by c-Myc is commonly mediated through transcriptional regulation of the cyclins and the cyclin-dependent kinase inhibitors, including cyclins D_1_, D_2_, and E and p21^WAF1/CIP1^ [26], [27] (http://www.myc-cancer-gene.org). Attenuating c-Myc expression specifically reduced cyclin D_1_ protein levels, but not cyclins D_2_ or E, in CD133+ cells (Figure 2B). Additionally, p21^WAF1/CIP1^ protein levels were upregulated in cancer stem cells depleted of c-Myc, whereas p53 levels were only moderately altered (Figure 2B). Quantitative real-time PCR showed a similar pattern of cyclin D_1_ and p21^WAF1/CIP1^ regulation at mRNA levels by c-Myc in CD133+ cells, suggesting c-Myc regulated these two genes at the level of transcription. In striking contrast, cyclin D_1_ and p21^WAF1/CIP1^ were minimally altered by knocking down c-Myc at either the mRNA or protein levels in CD133− cells. Notably, basal cyclin D_1_ levels were higher in CD133+ cells than matched CD133− cells, whereas cyclin D_2_ and cyclin E levels were higher in CD133− cells, suggesting that cyclin D_1_, but not cyclins D_2_ or E, is critical for the G_1_/S transition in CD133+ glioma cells. Taken together, these results suggest that c-Myc regulates cell cycle progression in glioma cancer stem cells, at least partially, through controlling expression of cyclin D_1_ and p21^WAF1/CIP1^.

**Figure 4:**
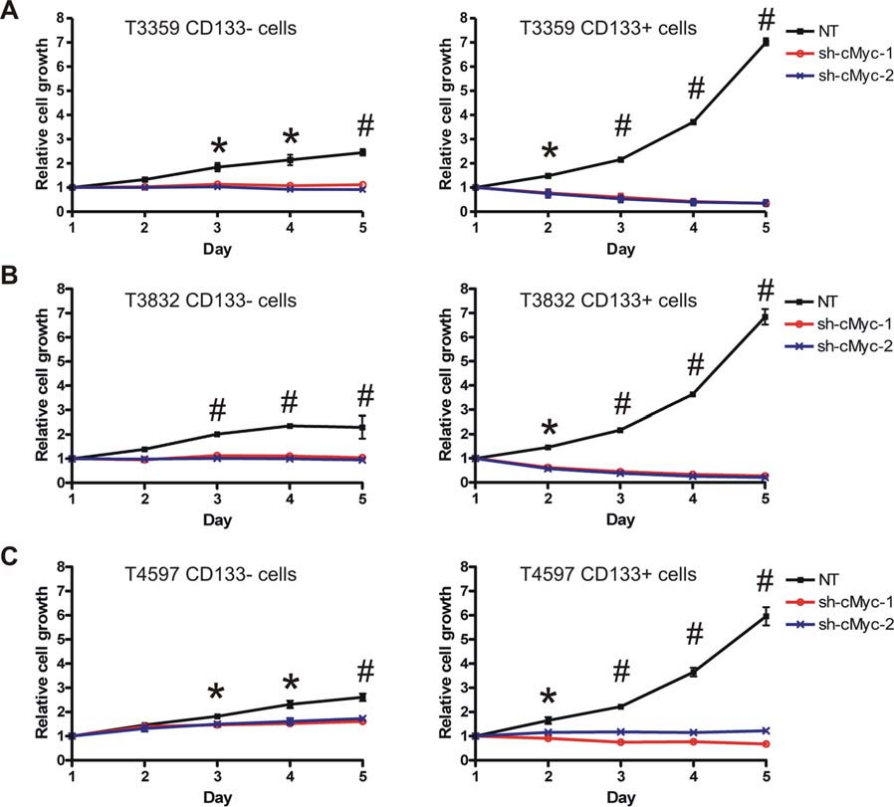
Depletion of c-Myc inhibits growth of glioma cancer stem cells. (A) T3359, (B) T3832, and (C) T4597 CD133− and CD133+ cells were isolated and infected as described. Cells were then plated in 96-well plates in triplicate at 5000 cells per well for CD133− cells or 1000 cells per well for CD133+ cells. Total viable cell numbers were then determined by the CellTiter-Glo Luminescent Cell Viability Assay (Promega) daily. *, p<0.05; #, p<0.001 with one-way ANOVA comparison of the control groups to the corresponding c-Myc knockdown groups on the same day.

Previous reports demonstrated that brain tumor stem cells isolated from human surgical biopsy specimens were actively proliferative *in vitro* [28], [29], but the numbers of matched CD133− tumor cells barely increased after a week of culture. Our data that the relatively high expression of c-Myc in glioma cancer stem cells is essential for their cell cycle regulation suggest that the growth of these cells may also require c-Myc activity. As demonstrated by growth curve assays, the total number of control CD133+ cells increased roughly six or seven fold over five days, whereas CD133+ cells depleted of c-Myc did not increase or decreased in number (Figure 4). Conversely, growth of the CD133− population was only moderately attenuated with c-Myc knockdown (Figure 4). These results suggest that c-Myc preferentially contributes to sustained growth of tumor initiating cells.

### Loss of c-Myc induces apoptosis in glioma cancer stem cells

**Figure 5:**
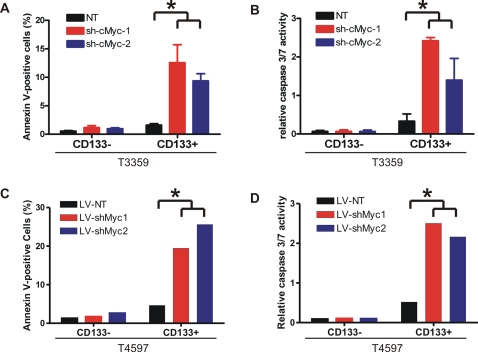
Depletion of c-Myc induces apoptosis in glioma cancer stem cells. (A, B) T3359 and (C, D) T4597 CD133− and CD133+ cells were isolated and infected as described. (A, C) Six days after infection, cells were trypsinized and quantified for apoptosis using the Annexin V-FITC Apoptosis Detection kit (Calbiochem). The percentage of FITC positive cells was determined by FACS analysis, and dead cells were excluded by propidium iodide staining. (B, D) These cells were also plated in 96-well plates at 5000 cells per well for CD133− cells and 1000 cells per well for CD133+ cells after infection and selection. Six days after infection, combined activities of caspase 3 and caspase 7 were determined by the Caspase 3/7 Luminescence Assay kit (Promega), and were normalized by the viable cell numbers determined by the CellTiter-Glo assays as described in Figure 4. *, p<0.05 with one-way ANOVA comparison of the control groups to the corresponding c-Myc knockdown groups.

c-Myc regulates not only cellular proliferation but also cellular survival. In concordance with a role in survival, we noted an accumulation of dead cells in the CD133+ fraction depleted of c-Myc expression during the growth curve experiments that was not present in controls (data not shown). Conversely, indications of cell death were limited in matched CD133− cells, regardless of c-Myc status, suggesting that loss of c-Myc might specifically induce apoptosis in CD133+ cells. The role of c-Myc in regulating apoptosis remains controversial. Previous reports have demonstrated that c-Myc promotes apoptosis in cancer cell lines and various normal cell types, whereas downregulation of c-Myc also leads to apoptosis under certain circumstances [30], [31], [32], [33]. To distinguish the anti-apoptotic or pro-apoptotic role of c-Myc in glioma cancer stem cells, we quantified apoptotic cell populations following knockdown of c-Myc. Annexin V staining revealed that depletion of c-Myc induced apoptosis in CD133+ glioma cells, whereas the control CD133+ cells contained a minimal apoptotic population (Figure 5A, C). In contrast, the CD133− population had only background staining of Annexin V regardless of c-Myc levels. Consistent with these data, the combined caspase 3/7 activity was elevated in CD133+ cells following c-Myc knockdown, but not in matched CD133− cells (Figure 5B, D). Taken together, these data suggest that c-Myc is a survival factor for glioma cancer stem cells.

### Loss of c-Myc impairs neurosphere formation by glioma cancer stem cells

**Figure 6:**
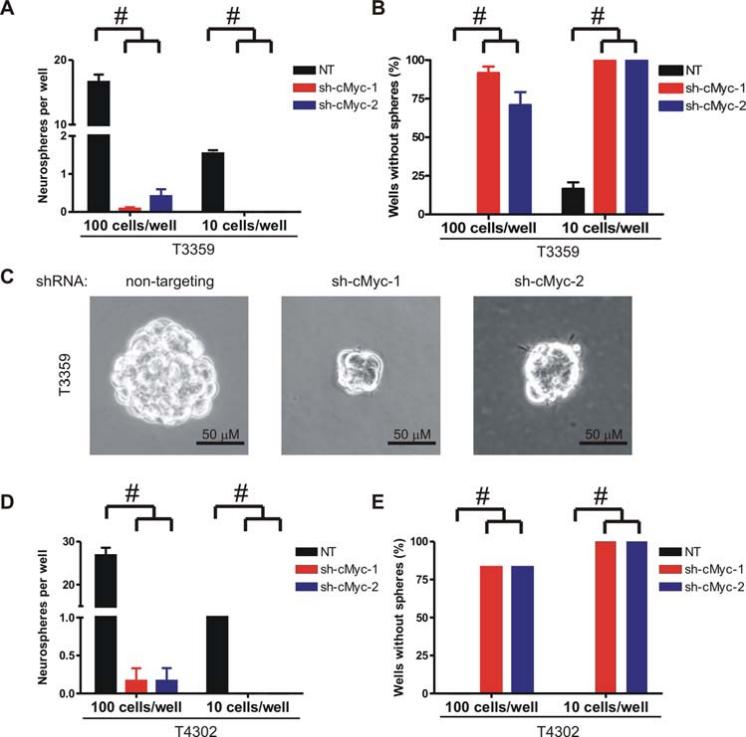
Depletion of c-Myc attenuates neurosphere formation by glioma cancer stem cells. (A) T3359 CD133+ cells were infected with lentivirus and selected as described. Cells were then plated at 100 or 10 cells per well in 24-well plates in the presence of 1 µg/ml puromycin. Eight wells were plated for each group. The numbers of neurospheres in each well were determined in seven days. Spheres that contained more than 20 cells were scored. (B) Percentage of wells without neurospheres formed was quantified. #, p<0.001 with one-way ANOVA comparison of the control groups to the corresponding c-Myc knockdown groups. (C) Representative photographs of neurospheres formed by cells expressing non-targeting shRNA or c-Myc specific shRNA (bars = 50 µm). (D, E) Similar results were demonstrated in T4302 CD133+ cells.

Sharing certain key characteristics of normal stem cells, cancer stem cells are capable of self-renewal, which allows sustained maintenance of this subpopulation and expansion of the whole tumor. Serial neurosphere formation assay has been utilized as a surrogate of the self-renewal capacity of neural stem cells [34], and was recently employed in the brain tumor stem cells [9], [28]. In primary neurosphere formation assays, approximately 15% of control CD133+ cells formed neurospheres, whereas very few spheres were formed by cells depleted of c-Myc (Figure 6A). Neurospheres developed in 100% of wells in the control group when plated at a density of 100 cells/well and in 80–85% wells when plated at 10 cells/well (Figure 6B). In contrast, neurospheres were formed by cells depleted of c-Myc expression in only 10% (shRNA-1) or 30% (shRNA-2) of wells when plated at a density of 100 cells/well, while no spheres were found when these cells were plated at 10 cells/well. Of note, neurospheres formed by cells with reduced c-Myc expression were markedly smaller than the spheres formed by the control cells (Figure 6C) and merely met the minimal criteria to be scored in our experiments. Lack of neurospheres formed by glioma cancer stem cells depleted of c-Myc suggests impaired self-renewal. However, assessing secondary neurosphere formation was precluded because very few spheres were generated in the knockdown groups in the primary assay and the neurospheres had limited viability. These studies therefore could not definitely determine if c-Myc expression is required for self-renewal of glioma cancer stem cells. However, it is expected that self-renewal of glioma cancer stem cells is inhibited by knockdown of c-Myc, based on our observations that glioma cancer stem cells failed to proliferate and underwent apoptosis upon knockdown of c-Myc.

### Knockdown of c-Myc inhibits the tumorigenic potential of glioma cancer stem cells

**Figure 7:**
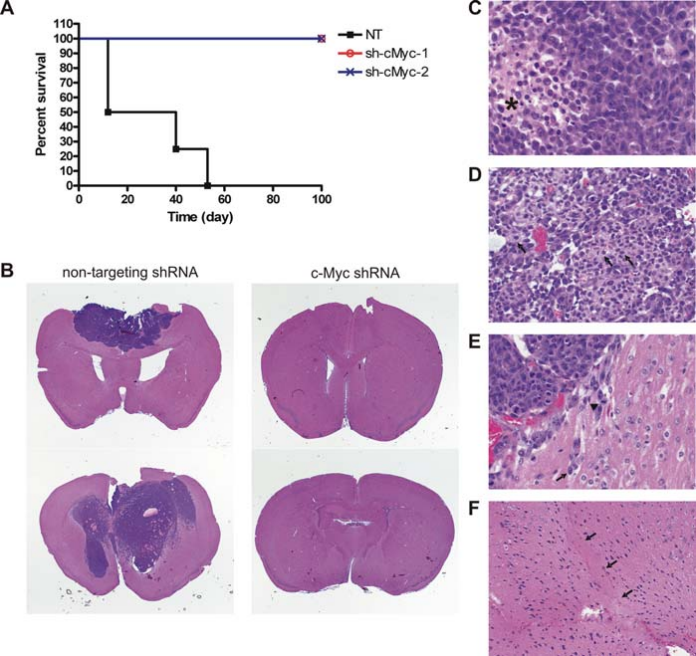
c-Myc knockdown abolishes xenograft tumor formation by glioma cancer stem cells. (A) T3359 CD133+ cells were infected and selected as described. After selection, cells were injected into brains of athymic BALB/c nu/nu mice (5000 cells per mouse). Four mice were injected for each group. Mice in the control group were sacrificed upon the development of neurologic signs. All the mice bearing c-Myc knockdown glioma cells did not develop neurologic signs and were sacrificed after 100 days without evidence of tumor. Kaplan-Meier survival curves are displayed. (B) Representative photographs of hematoxylin and eosin staining of intracranial xenograft tumors (10×). (C) Xenograft tissue of the control group composed of pleomorphic cells featuring high nuclear to cytoplasmic ratios, prominent nucleoli with minimal cytoplasm, brisk mitotic activity and central geographic necrosis (asterisk, 600×). (D) The control glioma xenograft exhibited focal areas of better differentiated tumor cells with relatively more eosinophilic cytoplasm and cells with eccentric cytoplasmic profiles suggestive of a gemistocytic appearance (arrows, 400×). (E) Xenograft tumor of the control group exhibits infiltration of tumor cells into the surrounding brain tissue along the margin. The mitotically active (arrowhead) infiltrating tumor cells exhibit high nuclear to cytoplasmic ratios and elongated fibrillar cytoplasm (arrow) (600×). (F) Mouse brain injected with T3359 cells expressing c-Myc shRNA showed no evidence of tumor at the needle injection site (arrows, 200×).

Based on the requirement of c-Myc activity for cell cycle progression, growth and survival, of CD133+ glioma cells in culture, we examined the role of c-Myc expression in their tumorigenic potential. CD133+ glioma cells were infected with non-targeting control lentivirus or lentivirus expressing c-Myc shRNA. Following puromycin selection, 5000 cells of each group were injected into the brains of nude mouse in quadruplicate. 100% of mice bearing control cells rapidly developed neurologic signs and displayed large tumors on histopathology composed of pleomorphic cells featuring high nuclear to cytoplasmic ratios, prominent nucleoli with minimal cytoplasm, brisk mitotic activity and central geographic necrosis, consistent with a high grade glial malignancy (Figure 7). In contrast, no mice injected with cells depleted of c-Myc expression developed signs and after 100 days demonstrated no evidence of neoplastic cells (Figure 7). Thus, c-Myc appears to be essential for cancer stem cells to form tumors.

## Discussion

The cancer stem cell hypothesis has engendered significant controversy, but the presence of heterogeneity of tumor cells in some cancers is well recognized. Tumor cells that display stem cell-like characteristics and initiate xenograft tumors have been purified from an increasing number of cancers, including leukemia [35], [36], brain [28], [29], breast [37], colorectal [38], [39], pancreatic [40], and head and neck cancers [41]. These cancer stem cells are functionally defined through assays of self-renewal and serial xenotransplantation assays in rodent models. Experiments demonstrate that the tumorigenic potential of cancer stem cells is at least several magnitudes higher than the bulk tumors. For example, glioma cancer stem cells enriched by selection of the CD133 surface antigen form orthotopic xenograft tumors with 500 cells in athymic nude mice, whereas 2×10^6^ CD133− cells cannot [9]. In line with the potent tumorigenicity of brain tumor stem cells, we demonstrated here that CD133+ glioma cancer stem cells highly expressed the c-Myc oncoprotein. FACS analysis demonstrated that at least half of CD133+ cells acutely dissociated from human surgical biopsy specimens had high levels of c-Myc, whereas the majority (>85%) of CD133− cells were c-Myc-low (Figure 1D). Similar co-expression of c-Myc and a stem cell marker, Nestin, was identified directly on human surgical biopsy specimen sections (Figure 1E). Notably, glioma cancer stem cells expressing c-Myc shRNA still retained residual levels of c-Myc protein (Figure 2B, lanes 5 and 6) that were higher than the c-Myc levels found in CD133− cells expressing non-targeting shRNA (Figure 2B, lane 1). Nonetheless, the reduced levels of c-Myc were incapable of supporting proliferation, growth, survival, and tumorigenesis of glioma cancer stem cells. A recent mouse model of T-cell lymphoma using conditionally expressed c-Myc also suggest that certain threshold level of c-Myc is required to maintain the tumor phenotype [42]. Collectively, our results highlight a necessary requirement of high c-Myc expression in glioma cancer stem cells.

Cancer stem cells have been proposed as slowly cycling cells, like their somatic stem cell counterparts [43]. Rapid expansion of tumors is then dependent on the fast dividing progenitor cells. The slow cell cycle may provide cancer stem cells a protective mechanism against certain therapeutic approaches that target rapid proliferating cells. For example, in human acute myelogenous leukemia, the quiescent leukemia stem cells are resistant to chemo-drugs that dependent on cell cycle [44]. Nevertheless, characteristics of cancer stem cells may not be necessarily uniform across different cancer types, and the biology of cancer stem cells may change throughout different stages of tumor development. In brain tumors, Singh and co-workers first demonstrated that only the CD133+ cancer stem cell population proliferate *in vitro* when acutely isolated from human surgical biopsy specimens [28], [29]. These results were similar to the highly proliferative cancer stem cells that were characterized in a RAS-induced zebrafish embryonal rhabdomyosarcoma [45]. In the present study, we determined that the CD133+ glioma cells contained a higher percentage of S phase cells and grew faster *in vitro* than matched CD133− cells (Figures 3 and 4). We also found that proliferation and growth of glioma cancer stem cells were severely impaired upon knockdown of c-Myc, but importantly, the cell cycle progression of CD133− glioma cells were relatively insensitive to loss of c-Myc. The transcriptional programs regulated by c-Myc remain poorly defined and dependent on cell state [26], [46], but include induction of cyclin D_1_ [47] and repression of the p21^WAF1/CIP1^ cyclin-dependent kinase inhibitor [48], [49]. Consistently, expression of cell cycle regulators downstream of c-Myc, including p21^WAF1/CIP1^ and cyclin D_1_, was altered in glioma cancer stem cells following c-Myc knockdown, but not in the CD133− cells. These data uncover a specific role of c-Myc and its downstream target genes in regulating proliferation and growth of the CD133+ glioma cancer stem cells.

Apoptosis can be induced by aberrant expression of certain oncogenes, such as c-Myc and E2F1, which may act as a “fail-safe” mechanism to eradicate potential cellular transformation [33], [50]. Apoptosis induced by c-Myc can be mediated through activation of the ARF-MDM2-p53 pathway, or by activating death receptors, such as CD95/Fas [33]. However, deregulation of c-Myc is common in human tumors and is an indicator of poor prognosis because tumors are well equipped with various anti-apoptosis mechanisms which can neutralize the apoptotic pressure introduced by c-Myc. Tumorigenesis in c-Myc transgenic mouse models is accelerated if combined with other anti-apoptosis genetic alterations, such as overexpression of Bcl-2 [51] or Bmi1 [52], or disrupting the ARF-MDM2-p53 pathway [53]. Further, it has been directly demonstrated that sustained c-Myc activity is required for tumor maintenance in a variety of conditional transgenic mouse models. These models show that inactivation of c-Myc almost inevitably results in tumor regression regardless of tumor type with concomitant growth inhibition, differentiation, and apoptosis (reviewed in [54]). In some cases, such as osteosarcoma [55] and skin tumors [56], brief inactivation of c-Myc is sufficient to induce sustained tumor regression. However, the dependence on c-Myc can be tumor-type specific. In certain conditional transgenic models, reactivation of c-Myc after transient disruption restores tumor growth [57], which may involve activation of dormant cancer stem cells. Alternatively, tumors may relapse in a portion of animals even in the absence of c-Myc activity, suggesting that cancer stem cells may have accumulated additional mutations to compensate loss of c-Myc [58], [59], [60]. The sustained tumor regression may be associated with complete eradication of cancer stem cells following inactivation of c-Myc, whereas cancer stem cells may survive and/or escape c-Myc dependence in recurrent tumors. Our study demonstrated a marked induction of apoptosis following c-Myc knockdown in the cancer stem cell population (Figure 4), and the cancer stem cells depleted of c-Myc expression failed to develop orthotopic xenograft tumors in nude mice (Figure 6). Of particular interest, survival of the non-stem glioma cells was not dependent on sustained expression of c-Myc. In a number of recent studies in other cancer models, targeting c-Myc expression impaired cellular proliferation and induced senescence [61], [62], [63]. These models may represent outcome of loss of c-Myc in more homogeneous cancer cell populations, particularly genetically engineered models driven by overexpression of c-Myc. Our study has important implications as targeting c-Myc has unique benefits specific to cellular compartments in models representing cellular heterogeneity. Although targeting c-Myc was sufficient to prevent tumor growth in our studies, the dichotomous effects of c-Myc inhibition that we have detected may support the need to simultaneously target the non-stem cell tumor populations to achieve clinical efficacy. Thus, c-Myc as a molecular target must be approached with sophistication as the majority of tumor cells may demonstrate limited therapeutic responses but a critical tumor population – the cancer stem cells – may be inhibited or killed to improve overall tumor control and decrease resistance to other therapies. Taken together, our study suggests that the proliferation, growth, and survival of glioma cancer stem cells are critically dependent on c-Myc expression and that targeting c-Myc pathways may significantly improve brain tumor therapy.

## Materials and Methods

### Cell isolation and culture

Primary glioma surgical biopsy specimens were obtained from patients undergoing resection for newly diagnosed or recurrent glioma in accordance with protocols approved by the Duke University Medical Center Institutional Review Board. Written consent to utilize excess tissue for research was obtained from each patient, and de-identified tissues were used for all studies. Cells were isolated from human glioma surgical specimens and cultured as previously described [3], [9]. Briefly, tumors were dissected, washed in Earle's balanced salt solution, digested with papain, titurated, and filtered. Red blood cells were lysed in diluted phosphate buffered saline solution (0.25×). Glioma cells were then cultured overnight in stem cell media (neurobasal media supplemented with B27 and epidermal growth factor and basic fibroblast growth factor (Invitrogen, Carlsbad, CA) at 20 ng/ml) prior to cell sorting for recovery of cellular surface antigens. The CD133− and CD133+ fractions were separated by magnetic sorting using the CD133 Microbead kit (Miltenyi Biotec, Auburn, CA). CD133− cells were maintained in Dulbecco's modified Eagle's medium (DMEM) with 10% fetal bovine serum (Invitrogen), but were cultured in stem cell media at least 24 hours prior to experiments to control differences in cell media. T3359, T3832, T4302 and T4597 were originally derived from human glioma surgical biopsy specimens and were maintained as subcutaneous xenografts in athymic BALB/c nude mice.

### Antibodies

The antibodies used were as follows: Mouse anti-c-Myc antibody (9E10), FITC-conjugated rabbit anti-c-Myc antibody (A14), mouse anti-cyclin D_1_ antibody (DCS-6), rabbit anti-cyclin D_2_ antibody (H-289), mouse anti-cyclin E antibody (13A3), mouse anti-p53 antibody (DO-1) were from Santa Cruz Biotechnology (Santa Cruz, CA); APC-conjugated mouse anti-CD133 antibody (AC133-1) was from Miltenyi; goat anti-Olig2 antibody was from R&D Systems (Minneapolis, MN); mouse anti-p21^WAF1/CIP1^ antibody was from Cell signaling Technology (Danvers, MA); mouse anti-actin antibody was from Millipore (Billerica, MA).

### Immunofluorescent staining

Freshly frozen human glioma surgical biopsy samples were processed and 10 micron sections were mounted on glass slides in accordance with the Duke University Medical Center Institutional Review Board. Prior to staining, the sections were fixed in 4% paraformaldehyde at room temperature for 10 minutes, boiled in 10 mM sodium citrate solution (pH = 6.0) for 10 minute, and were blocked in 10% normal goat serum (Sigma-Aldrich, St. Louis, MO) and 0.1% Triton X-100 for 30 minutes at room temperature. Sections were incubated with primary antibodies at 4°C overnight and secondary antibodies at room temperature for 45 minutes, and then were counterstained with Hoechst 33342 (Invitrogen). For fluorescence imaging, confocal z-stacks were taken by a 63× water immersion objective lens on a Leica SP-5 microscope using sequential scans (blue-red, green). The following antibodies were used: mouse anti-c-Myc (Santa Cruz, 1∶50), rabbit anti-Nestin (Abcam, Cambridge, MA, 1∶100); goat anti-mouse IgG_1_ Alexa 488 (Invitrogen, 1∶200), goat anti-rabbit IgG Alexa 568 (Invitrogen, 1∶200).

### Lentivirus production

The lentiviral vectors directing expression of shRNA specific to c-Myc (TRCN0000039640 and TRCN0000039641) or a non-targeting shRNA (SHC002) (Sigma-Aldrich) were co-transfected with the packaging vectors psPAX2 and pCI-VSVG (Addgene, Cambridge, MA) into 293FT cells by lipofectamine 2000 (Invitrogen) to produce virus. Two days following transfection, viral supernatants were collected, filtered, and concentrated by ultracentrifugation at 100,000 g for 3 hours.

### FACS analysis

Glioma cells isolated from surgical biopsy specimens were fixed in 4% paraformaldehyde, and permeablized in 0.1% Triton X-100 for 10 minutes. Cells were then washed twice in PBS and labeled with APC-conjugated CD133 antibody (AC133, 1∶11) and FITC-conjugated c-Myc antibody (A14, 1∶50) for 1 hour at room temperate. Cells were then washed once in PBS and sorted.

### Real-time PCR

Total RNA was prepared using the RNeasy kit (Qiagen, Valencia, CA), and reverse transcribed into cDNA by iScript cDNA synthesis kit (BioRad, Hercules, CA). Real-time PCR was performed on an ABI 7900HT system using SYBR-Green Mastermix (SuperArray, Frederick, MD). PCR products were verified by melting curves and were also run on a 2% agarose gel to confirm the appropriate size. The threshold cycle (*C*_T_) values for each gene were normalized to expression levels of β-Actin and HPRT1 (hypoxanthine phosphoribosyltransferase 1). The primers used were as follows: β-Actin, forward 5′-AGAAAATCTGGCACCACACC-3′; reverse 5′-AGAGGCGTACAGGGATAGCA-3′; HPRT1, forward 5′-TGACCTTGATTTATTTTGCATACC-3′; reverse 5′-CGAGCAAGACGTTCAGTCCT-3′; c-Myc, forward 5′-TCAAGAGGCGAACACACAAC-3′; reverse 5′-GGCCTTTTCATTGTTTTCCA-3′; cyclin D1, forward 5′-CCGCTGGCCATGAACTACCT-3′; reverse 5′-ACGAAGGTCTGCGCGTGTT-3′; cyclin D2, forward 5′-TGGAGCTGCTGTGCCACG-3′; reverse 5′-GTGGCCACCATTCTGCGC-3′; p21^WAF1/CIP1^, forward 5′-TCACTGTCTTGTACCCTTGTGC-3′; reverse 5′-GGCGTTTGGAGTGGTAGAAA-3′.

### Growth Curve and Neurosphere formation assay

Both CD133− and CD133+ glioma fractions of glioma cells were infected by the control lentivirus or lentivirus directing expression of c-Myc shRNA and were selected with 1 µg/ml puromycin for 2 days. 5000 CD133− cells or 1000 CD133+ cells were plated in 96-well plate in triplicate. Cell number was measured for 5 consecutive days using the CellTiter-Glo assay kit (Promega, Madison, MI). Additionally, 100 or 10 CD133+ cells were seeded per well in 24-well tissue culture plates. Eight wells were plated for each group. Seven days after plating, neurospheres containing more than 20 cells were scored.

### Intracranial xenograft formation assay

5000 T3359 CD133+ cells lentivirally infected and selected for expression of the puromycin marker were implanted into brains of athymic BALB/c nude mice under a Duke University Institutional Animal Care and Use Committee–approved protocol. Mice were maintained for 100 days or until development of neurologic signs. Brains of euthanized mice were collected, fixed in 4% paraformaldehyde, paraffin embedded, sectioned, and subjected to hematoxylin and eosin staining.
